# The Complete Mitochondrial Genome of the Siberian Scoter *Melanitta stejnegeri* and Its Phylogenetic Relationship in Anseriformes

**DOI:** 10.3390/ijms251810181

**Published:** 2024-09-22

**Authors:** Huimin Chen, Yaqin Chen, Zhenqi Wang, Dawei Wu, Pan Chen, Yanhong Chen

**Affiliations:** 1 The Anhui Provincial Key Laboratory of Biodiversity Conservation and Ecological Security in the Yangtze River Basin, College of Life Sciences, Anhui Normal University, Wuhu 241000, China; huiminchen2022@163.com (H.C.); 17870865324@163.com (Y.C.); 2 College of Life Sciences, Nanjing Forestry University, Nanjing 210037, China; arkia0205@njfu.edu.cn (Z.W.); wudawei@njfu.edu.cn (D.W.)

**Keywords:** *Melanitta stejnegeri*, mitogenome, phylogenetic analysis

## Abstract

The Siberian Scoter (*Melanitta stejnegeri*) is a medium sea duck distinct from *M. deglandi* due to the absence of hybridization and differences in morphological characteristics. However, knowledge of its phylogenetic relationships within Anseriformes is limited due to a lack of molecular data. In this study, the complete mitogenome of *M. stejnegeri* was firstly sequenced, then annotated and used to reconstruct the phylogenetic relationships of 76 Anseriformes species. The complete mitogenome of *M. stejnegeri* is 16,631 bp and encodes 37 typical genes: 13 protein-coding genes, 2 ribosomal RNAs, 22 transfer RNAs, and 1 non-coding control region. Its mitogenome organization is similar to that of other Anseriformes species. The phylogenetic relationships within the genus *Melanitta* are initially clarified, with *M. americana* at the base. *M. stejnegeri* and *M. deglandi* are sister groups, clustering with *M. fusca* and *M. perspicillata* in order. Phylogenetic analysis suggests that *Mareca falcata* and *M. strepera* are sister groups, differing from previous studies. Results firstly indicate that *Clangula hyemalis* and *Somateria mollissima* are sister groups, suggesting a potentially skewed phylogenetic relationship may have been overlooked in earlier analyses relying solely on mitochondrial genomes. Our results provide new mitogenome data to support further phylogenetic and taxonomic studies of Anseriformes.

## 1. Introduction

The mitochondrial genome (mitogenome) is the only extranuclear genome in animal cytoplasm, and is characterized by several features: the abundance of mitochondria in cells, maternal inheritance, absence of introns, and higher evolutionary rates [1,2]. Mitochondrial (mt) genes and genomes are major sources of data for evolutionary studies in birds, and complete mitogenomes have been used to unveil the phylogenetic relationships among major orders [3]. The mitogenomes of vertebrates are double-stranded circular molecules, typically 16–18 kbp in size, containing a consistent set of 13 protein-coding genes (PCGs), 2 ribosomal RNAs (rRNAs), 22 transfer RNAs (tRNAs), and 1 non-coding control region (D-loop) [4]. Five distinct mitochondrial gene orders have been described in birds, known as ancestral avian, remnant control region (CR), duplicate CR, and duplicate *trnT*-CR [5].

The Siberian Scoter (*Melanitta stejnegeri*) is a medium-sized sea duck that feeds on molluscs, crustaceans, worms, echinoderms, amphipods, isopods, small fish, and insects, and may also consume plant material on its breeding grounds (e.g., leaves and shoots) [6]. It belongs to the Anatidae family, *Melanitta* genus, which has only six species, and breeds in eastern Asian Russia. It is widely distributed along the coasts of Asia, from Japan to China, inhabiting forests, grasslands, wetlands (inland), and marine neritic zones [7]. The IUCN Red List of Threatened Species has assessed it as a species of Least Concern, despite a decreasing population trend. This classification is due to its extremely large range and population, which do not meet the thresholds for classification as Vulnerable based on the range size criterion and the population trend criterion [6]. Additionally, this species has been recognized as distinct from *M. deglandi* by the IOC World Bird List (v9.2) based on the absence of hybridization and differences in morphological characteristics [7]. However, the complete mitogenome data of *M. stejnegeri* remains unknown, limiting its ecological and evolutionary analysis. The order Anseriformes includes 3 families, 8 tribes, and more than 150 species [8]. Previous analyses of phylogenetic relationships in Anseriformes or Anatidae species remain uncertain due to the limited availability of published mitochondrial gene sequences [8,9].

In this study, we firstly obtained the complete mitochondrial genome sequence of *M. stejnegeri* through high-throughput sequencing, analyzed the structural organization and characteristics of its mitogenome, and reconstructed its phylogenetic relationships in Anseriformes based on the acquired mitogenome and genome date of 76 avian species. The revealed data on the mitochondrial genome can be employed to generate molecular markers, evaluate genetic diversity, and help understand the phylogenetic and evolutionary relationships of the *Melanitta* genus. Our results provide more reliable molecular biological data for future phylogenetic and taxonomic studies of Anseriformes.

## 2. Results and Discussion

### 2.1. Mitogenome Organization

**Figure 1:**
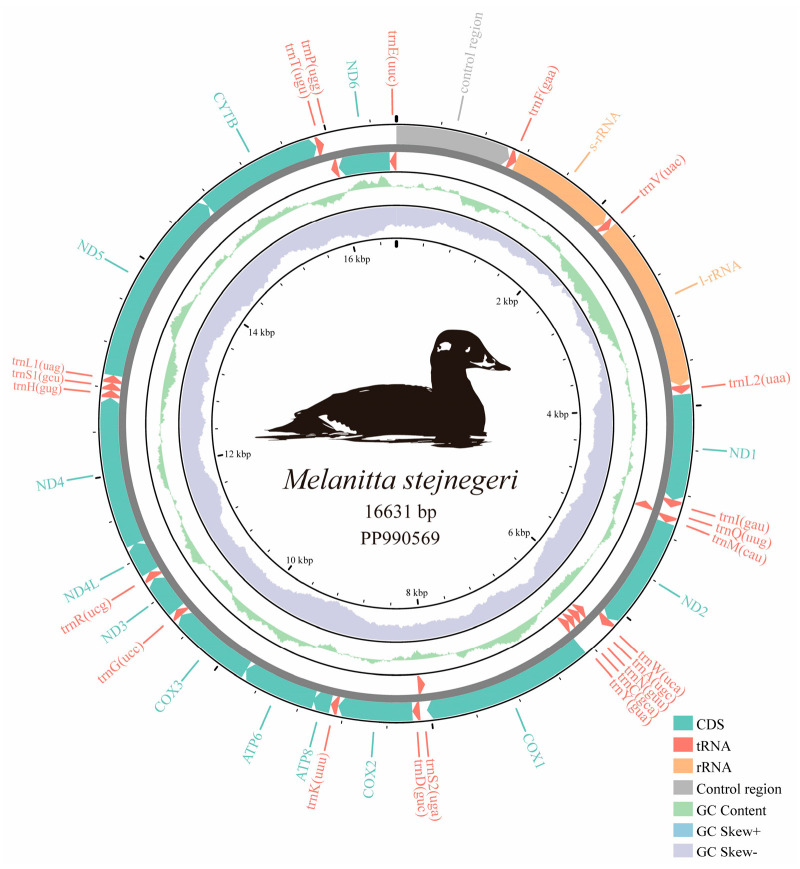
Gene map of the *Melanitta stejnegeri* mitogenome. Arrows indicate the orientation of gene transcription. The outer circle shows the arrangement of genes: outer genes on the heavy strand (H-strand) and inner genes on the light strand (L-strand). Protein-coding genes (PCGs) are represented by green arrows, tRNAs by red arrows, rRNAs by orange arrows, and the control region by gray arrows. The green ring indicates the GC content, with outward and inward peaks indicating above- and below-average GC content, respectively. The purple–blue ring represents the GC skew [(G − C)/(G + C)], with purple for values between −1 and 0 and blue for values between 0 and 1. Ticks in the inner circle indicate the sequence length.

A total of 31,876,685 raw reads were generated by next-generation sequencing on the Illumina platform. The length of the avian mitogenome ranges from about 16.3 kb to 20 kb [10]. The complete mitogenome of *M. stejnegeri* is 16,631 bp long, larger than most Anseriformes species, and the sequence has been submitted to GenBank with the accession number PP990569. The entire mtDNA of *M. stejnegeri* comprises 13 PCGs, 22 tRNA genes, 2 rRNA genes (12S rRNA and 16S rRNA), and 1 D-loop (1071 bp) located between *trnE* and *trnF*. The mitochondrial gene order of *M. stejnegeri* follows the ancestral avian gene order (Figure 1) [5]. The 13 PCGs consist of 7 subunits of NADH dehydrogenase (ND1, ND2, ND3, ND4L, ND4, ND5, and ND6), 3 subunits of cytochrome oxidase (COX1, COX2, and COX3), 1 subunit of cytochrome b (CYTB), and 2 subunits of ATP synthase (ATP6 and ATP8). Nine genes, including *ND6* and 8 tRNAs (*trnQ*, *trnA*, *trnN*, *trnC*, *trnY*, *trnS2*, *trnP* and *trnE*) are located on the light strand (L-strand), whereas the other 28 genes are found on the heavy strand (H-strand) (Figure 1 and Table S1).

The parameters of A+T content, AT skew, and GC skew are commonly used to study the base composition pattern of mitogenomes [11]. The overall base composition of the mtDNA of *M. stejnegeri* is 28.72% A, 21.57% T, 33.52% C, and 16.2% G. The percentage of A and T (50.29%) is slightly higher than that of G and C (49.71%) (Table 1). The overall AT skew and GC skew in the *M. stejnegeri* mitogenome are 0.1422 and −0.3483, respectively. The GC skew is slightly negative (−0.3605 to −0.0029), indicating a higher occurrence of C than G. The overall AT skew is slightly positive (0.0490 to 0.2641), suggesting a higher content of A than T. These results demonstrate that the genome sequence skews away from T and G in favor of A and C, consistent with other Anseriformes species, supporting a slightly specific bias towards A and C in Anseriformes species [8,12].

### 2.2. Protein-Coding Genes

The total length of all *M. stejnegeri* PCGs is 11,407 bp, accounting for 68.59% of the entire mitogenome, which translates to 3791 amino acid-coding codons, excluding stop codons (33 bp) (Table 1). Ten PCGs begin with an ATG start codon, while three other genes (*COX1*, *COX2*, and *ND5*) begin with GTG (Table S1). Regarding stop codons, seven PCGs (*ATP6*, *ATP8*, *ND3*, *ND4L*, *ND5*, *COX2*, and *CYTB*) end with TAA, two (*ND1* and *COX1*) with AGG, and *ND6* ends with TAG, while three (*ND2*, *ND4*, and *COX3*) have an incomplete stop codon (T-), presumably completed by post-transcriptional polyadenylation [13]. Additionally, *ATP8* and *ATP6* share 10 nucleotides, *ATP6* and *COX3* overlap by 1 nucleotide, *ND4L* and *ND4* share 7 nucleotides, and *ND5* and *CYTB* have an interval of 2 nucleotides.

**Figure 2:**
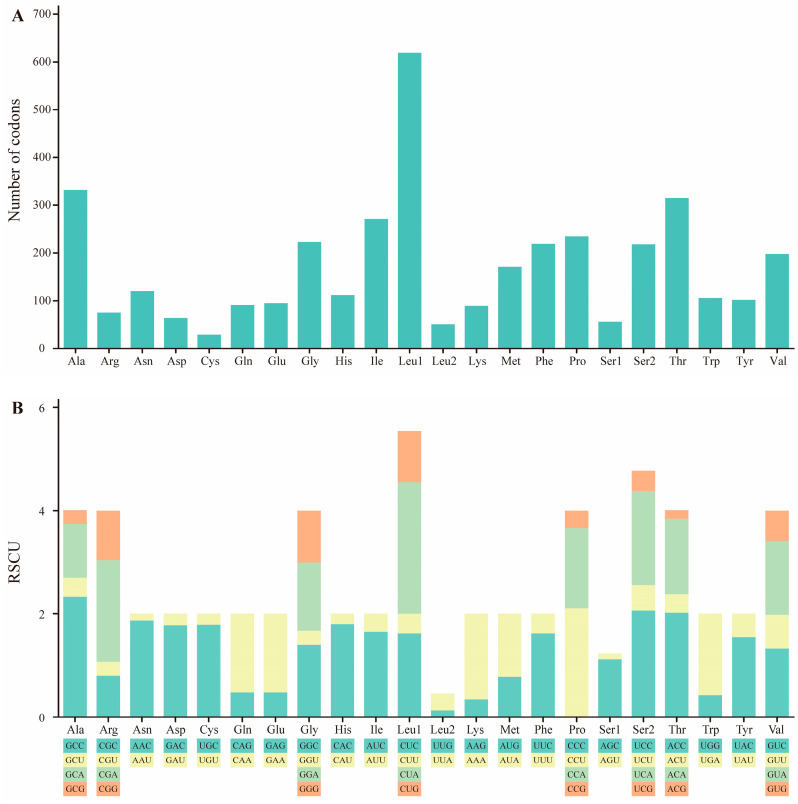
(**A**) Codon distribution of the *M. stejnegeri* mitogenome. Numbers on the *Y*-axis refer to the total number of codons and codon families are listed on the *X*-axis. (**B**) The relative synonymous codon usage (RSCU) of *M. stejnegeri*. Codons are shown on the *X*-axis, and RSCU values are shown on the *Y*-axis.

The total number of codons in PCGs is 3801. Codons encoding Cys are the rarest, while those encoding Leu1 (CUX), Thr, and Ala are the most frequent (Figure 2A). The genetic codon bias for the *M. stejnegeri* mitogenome is illustrated by RSCU (Table S2). The base composition of PCGs reveals a skew in the use of synonymous codons for most amino acids and a preference for using the A or C nucleotides in the third codon position, such as the GCC codon for Ala (Figure 2B). Additionally, nucleotide prevalence in the third position of the codon varies across taxa [14].

### 2.3. Ribosomal and Transfer RNA Genes

The 12S rRNA and 16S rRNA genes are 996 bp and 1602 bp in length, with A+T contents of 50.20% and 54.12%, respectively. Similar to most vertebrates, these rRNA genes are situated between *trnF* and *trnL2* and are isolated by *trnV*.

**Figure 3:**
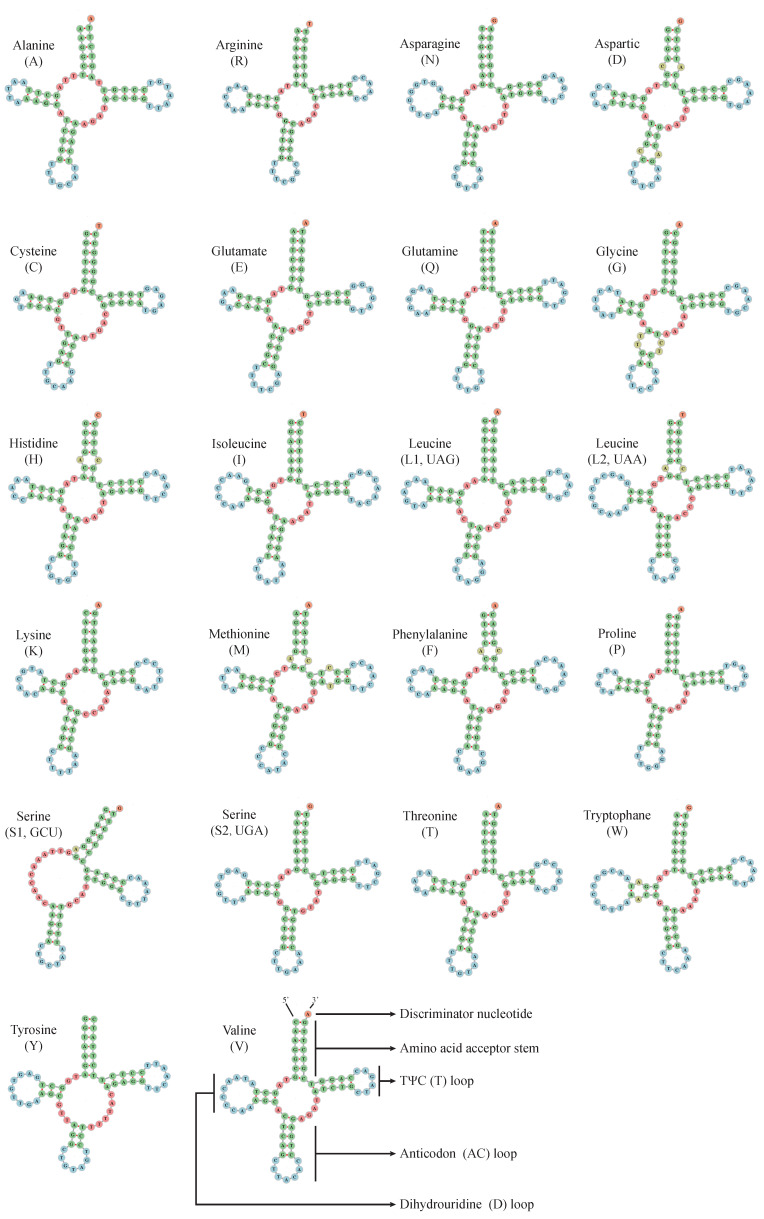
Secondary structures of the 22 transfer RNA genes of *M. stejnegeri*.

The *M. stejnegeri* mitogenome contains 22 tRNAs, with a total length of 1546 bp for all tRNAs. Each tRNA gene varies in size from 66 bp (*trnC* and *trnS1*) to 76 bp (*trnW*) (Table S1). The A+T content of the 22 tRNAs is 55.50%, ranging from 40.91% (*trnC*) to 69.01% (*trnQ*) (Table 1 and Table S3). All tRNA genes exhibit the cloverleaf secondary structure with normal base pairing, except for *trnS1*, which lacks the entire dihydrouridine arm, a characteristic commonly observed in vertebrate tRNA genes (Figure 3) [15]. The absence of this arm in *trnS1* may play a functional role in the structural compensation mechanism among other structures [16]. The mt-*trnS*1 loses its tRNA core domain, resulting in the absence of identity elements in *trnS1* due to the lack of the dihydrouridine arm. However, studies have indicated that the human mitochondrial *trnS1* synthetase has evolved to specifically bind to the specific acceptor stem of *trnS1* through its unique helical arm to charge the tRNA [17,18].

### 2.4. Non-Coding Region

The major D-loop, or mitochondrial CR, consists of hyper-variable non-coding sequences and regulates the replication and transcription of mtDNA [19]. The D-loop of *M. stejnegeri* is located between *trnE* and *trnF*, with a length of 1071 bp (Table S1). The base composition of the D-loop is 26.89% A, 33.05% C, 23.81% T, and 16.25% G (Table 1). The A+T content (50.70%) is higher than the C + G content (49.30%) and the overall genome A+T content (50.29%). The AT skew and GC skew values of the D-loop are 0.0608 and −0.3409, respectively. A study of D-loop sequences in 68 avian species suggested that the distribution of variable nucleotide positions within the D-loop was genus-specific and not dependent on the level of divergence [20].

### 2.5. Analysis of Phylogenetic

**Figure 4:**
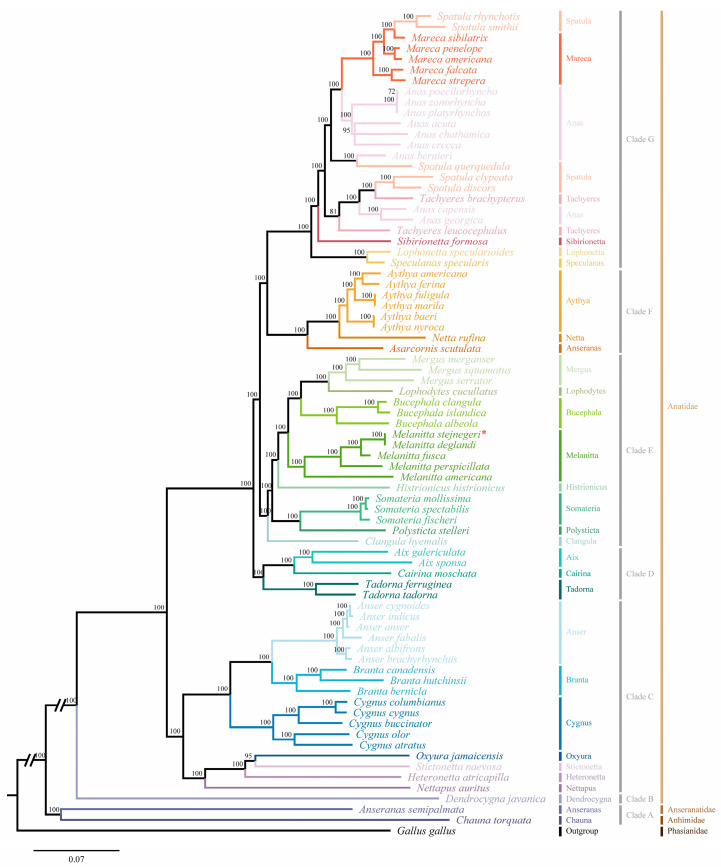
Phylogenetic relationships of 76 Anseriformes species reconstructed using the Bayesian Inference (BI) method based on 13 mitochondrial protein-coding genes. Numbers on the nodes represent the values of the Bayesian posterior probabilities.

Based on all accessible sequence data published on the NCBI, 13 PCGs from 76 Anseriformes species and chicken were acquired, except for the *ND1* of *Cygnus buccinator* and *ND3* of *Lophonetta specularioides* (Table S4). The best-fit model of GTR+I+G was selected by the MrModeltest 2.4 to reconstruct the phylogenetic relationship of 76 avian species in Anseriformes. Anseriformes species are relatively clearly classified at the family level but not at the subfamily and genus levels. Phylogenetic analysis based on the complete mitogenome could strongly assist in the classification of birds [8]. Results showed that the tree could be divided into seven clades (Figure 4). The phylogenetic relationships of 76 avian species, reconstructed using Bayesian inference (BI) and maximum likelihood (ML) methods, are generally consistent. However, the phylogenetic relationship of *Tachyeres leucocephalus* is different, and the Bayesian posterior probability value for this species is higher (Figure 4 and Figure S1). Clade A consists of Anseranatidae and Anhimidae, while the other six clades consist of 74 avian species belonging to 29 genera in Anatidae. Clade B contains just one species, *Dendrocygna javanica*, in the genus *Dendrocygna*. Clade C contains seven genera: *Nettapus*, *Heteronetta*, *Stictonetta*, *Oxyura*, *Cygnus*, *Branta*, and *Anser*. Clade D includes *Tadorna*, *Cairina*, and *Aix*. Clade E is a larger branch that includes eight genera: *Clangula*, *Polysticta*, *Somateria*, *Histrionicus*, *Melanitta*, *Bucephala*, *Lophodytes*, and *Mergus*. Clade F includes *Anseranas*, *Netta*, and *Aythya*. The last clade, Clade G, contains six genera: *Speculanas*, *Lophonetta*, *Sibirionetta*, *Tachyeres*, *Anas*, *Spatula*, and *Mareca*. Additionally, Clades D to G form a larger branch within Anatidae. The results suggest that *Heteronetta atricapilla* is a sister to *Oxyura jamaicensis* and *Stictonetta naevosa*, and *Mareca falcata* and *M. strepera* are sister species, which is slightly different from a previous study [8]. The results also support that *L. specularioides* and *Speculanas specularis* are sister groups to each other [9]. Furthermore, the phylogenetic relationships within the genus *Melanitta* are clarified initially, with *M. americana* located at the base of this genus. Meanwhile, *M. stejnegeri* and *M. deglandi* are sister groups, which subsequently cluster with *M. fusca* and *M. perspicillata* in order, and five *Melanitta* species form a closely related clade. Notably, *Anas capensis* and *A. georgica* do not group with other *Anas* species but with *Tachyeres* species. Similarly, *Spatula rhynchotis*, *S. smithii*, and *S. querquedula* do not group with *S. clypeata* or *S. discors*. Considering that the majority of PCGs of *S. querquedula* acquired are partial genes, higher-quality sequenced data might make the results for this species more reliable. Additionally, traditional species trees are inferred using morphological characters, such as morphological, physiological, or behavioral traits, but these may face challenges due to phenotypic plasticity and could lead to incorrect identifications [21]. A study has shown that, on average, morphological characters experience much more convergence than amino acid sites, and morphological convergent evolution might confound phylogenetic reconstruction [22]. 

**Figure 5:**
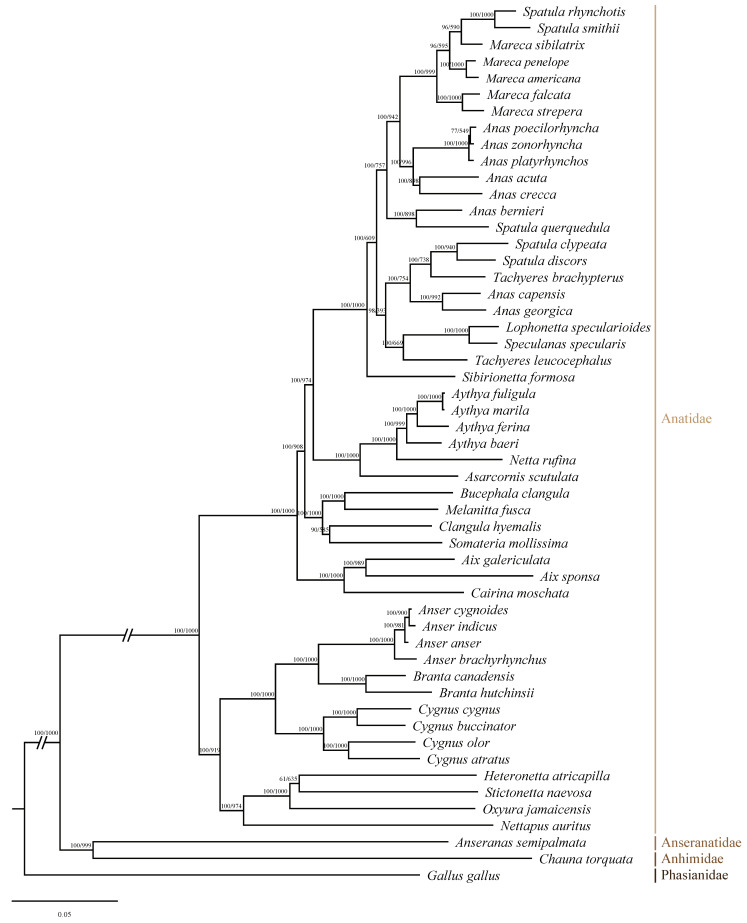
Phylogenetic relationships of 52 Anseriformes species and one outgroup reconstructed based on 13 PCGs and 3 nuclear genes. Above the nodes, the left value represents the Bayesian posterior probability, and the right value represents the bootstrap proportion.

Considering the maternal inheritance patterns of mitochondrial genomes and the selective pressures on them, which may result in incomplete or skewed phylogenetic relationships, three commonly used nuclear genes (*RAG-1*, *c-MOS*, and *c-MYC*) were selected and obtained from 53 avian species, including one outgroup, to reconstruct their phylogenetic relationships based on three nuclear genes and 13 PCGs (Table S4). The phylogenetic results were then compared with previous phylogenetic relationships reconstructed solely based on 13 PCGs. Results indicated that both BI and ML analyses yielded the same inferred phylogenetic relationships, differing only in branch lengths and levels of support (Figure 5). Notably, the phylogenetic relationships of five species (*A. capensis*, *A. georgica*, *S. rhynchotis*, *S. smithii*, and *S. querquedula*) did not cluster with other species of their respective genera, which is consistent with the results based on the 13 PCGs. The findings ruled out the effectiveness of relying solely on mitochondrial genes, supporting our view that inconsistencies in the classification based on morphological characteristic and molecular phylogenetic relationships among these five species necessitate further study. The results confirmed that *Mareca falcata* and *M. strepera* are sister groups, which differs from previous studies [8]. Furthermore, it was found that the phylogenetic relationships of six other avian species (*L. specularioides*, *S. specularis, Clangula hyemalis, Somateria mollissima, H. atricapilla, S. naevosa,* and *O. jamaicensis*) are inconstant with the phylogenetic results based on 13 PCGs. Results showed that *L. specularioides* and *S. specularis* are sister groups clustering with *T. leucocephalus*, which contradicts our findings based on the 13 PCGs but aligns with previous studies based on 3 mitochondrial genes (*CYTB*, *ND2*, and *COXI*) [23]. Notably, *C. hyemalis* and *S. mollissima* were initially identified as sister groups, which is inconsistent with earlier studies [8,23]. Previous research has suggested that Anseriformes species with different feeding habits experience varying selective pressures on their mitochondrial genomes [24]. Given that previous study on the phylogenetic relationships of Anseriformes species primarily focused on mitochondrial genes, and considering that *C. hyemalis* and *S. mollissima* mainly feed on invertebrates, similar dietary habits might have caused similar selective pressure on their mitochondrial genomes. Consequently, skewed phylogenetic relationships may exist in earlier studies that solely relied on mitochondrial genomes [8,24]. Finally, it was observed that *H*. *atricapilla* and *S*. *naevosa* initially cluster together and subsequently cluster with *O*. *jamaicensis*, which is consistent with previous studies but not with the results based on the 13 PCGs [8]. Further study on the classification of Anseriformes species using integrative taxonomy are needed. Although this study has not fully resolved the classifications within Anatidae, it has provided valuable reference material for the taxonomy of Anseriformes.

## 3. Materials and Methods

### 3.1. Sample Collection and Mitogenome Sequencing

A naturally deceased Siberian Scoter was found at Dafeng Milu National Nature Reserve in Jiangsu Province, China (32°56′–33°36′ N, 120°42′–120°51′ E). The sample was preserved in 95% ethanol and stored in a deep freezer for future use. Total genomic DNA was extracted from muscle tissues and sequenced using Illumina NovaSeq 6000 by Beijing BerryGenomics Co., Ltd. (Beijing, China). The raw data consisted of 31,876,685 reads and underwent assessment and quality control following default rules using Berry FastQC 0.12.0 [25]. The paired reads were filtered out if the content of unknown bases (N) in single-end reads exceeded 3, or if the number of low-quality nucleotides (Q-value < 5) exceeded 20% of the read length. Additionally, when removing the adapter sequence, the adapter must match at least 8 bp.

### 3.2. Assembly, Annotation, and Analysis of the Mitogenome

The mitogenome was assembled using MitoZ [26]. The tRNA genes were identified and annotated against vertebrate mitogenomic codes using MITOS WebServer [27], and each tRNA gene was checked manually. Subsequently, secondary structures of the tRNA genes were predicted and visualized using tRNAscan-SE 2.0 and ViennaRNA Web Services independently [28,29]. The initiation and termination codons of the 13 PCGs were identified in the mitogenome of *M. stejnegeri* using the open reading frame (ORF) finder (https://www.ncbi.nlm.nih.gov/orffinder/, accessed on 25 May 2024) with settings for the vertebrate mitochondrial genetic code, and were manually adjusted with reference to the annotated mitogenome of *A. platyrhynchos* (NC_009684). 

Base compositions were calculated, and relative synonymous codon usage (RSCU) values were analyzed using MEGA 11 [30]. Composition skew was estimated using the formulas “AT-skew = (A − T)/(A + T)” and “GC-skew = (G − C)/(G + C)” [31]. Furthermore, a graphical map of the *M. stejnegeri* mitogenome was generated using Proksee [32].

### 3.3. Phylogenetic Analysis

To determine the phylogenetic relationship of *M. stejnegeri* and other avian species in Anseriformes, 13 PCGs from 76 Anseriformes species and chicken were used to reconstruct phylogenetic relationships. The data were derived from 56 avian mitogenome sequences, 21 avian genome sequences, and 12 gene nucleotide sequences. Additionally, the mitogenome sequence of the chicken was set as the outgroup. These sequences were downloaded from the National Center for Biotechnology Information (NCBI) database (https://www.ncbi.nlm.nih.gov/, accessed on 30 May 2024; Table S4). To identify the 13 PCGs from the 21 avian genomes, the protein sequences of the 13 PCGs from human, mouse, mallard, chicken, lizard, frog, and zebra fish were used as queries for tblastn searches with a cutoff e-value of 1 × 10^−10^. Subsequently, the putative PCGs sequences were extended by 100 bp in the 5′ and 3′ directions for further analysis to determine the nucleotide sequences using the online tool GeneWise (https://www.ebi.ac.uk/jdispatcher/psa/genewise, accessed on 10 June 2024). Gene trees based on all acquired putative 13 PCGs were constructed using MEGA 11 to identify the best-fit sequences individually. The PCGs of chicken were used as a reference, and all 13 identified PCGs containing sequences of *M. stejnegeri* were translated into proteins and aligned individually using MEGA 11, and then manually checked before being concatenated through DAMBE [33]. 

Phylogenetic relationships of 77 avian species were reconstructed using both BI and ML approaches. The BI phylogenetic tree was generated with MrBayes 3.2.7 [34] over 5 million generations until the average standard deviation of split frequencies was less than 0.01, following the selection of the best-fitting substitution model GTR+I+G by the MrModeltest 2.4 [35]. The ML tree was constructed with 1000 bootstraps based on the optimal model determined by jModelTest 2.1.10 [36]. Subsequently, the two phylogenetic trees were visualized using Figtree 1.4.4 and compared [37]. 

To compare the phylogenetic results of Anseriformes species reconstructed based solely on mitochondrial genes with results based on both nuclear and mitochondrial genes, three nuclear genes (*RAG-1*, *c-MOS*, and *c-MYC*) were utilized to analyze phylogenetic evolution. These genes were obtained from 53 avian genomes, including one outgroup, using previously described methods. Subsequently, the nuclear gene sequences of the 53 avian species were aligned and concatenated. The best-fitting substitution model GTR+I+G was selected for the nuclear genes using MrModeltest 2.4. Finally, the nuclear and mitochondrial characters were concatenated, and the phylogenetic relationships of the 53 avian species were reconstructed using BI and ML methods individually.

## 4. Conclusions

In this study, we first sequenced and annotated the mitogenome of *M. stejnegeri*, which is 16,631 bp in length and larger than most Anseriformes species. The mitogenome consists of 13 PCGs, 22 tRNA genes, 2 rRNA genes, and 1 D-loop (1071 bp), with the genes ordered according to the ancestral avian gene order. A positive AT skew consistent with other Anseriformes species was found, supporting a slight bias towards A and C in Anseriformes. The 13 PCGs account for 68.59% of the whole mitogenome, most of which are encoded by the H-strand. ATG and TAA are the most frequent start and stop codons, respectively. The RSCU values for the 13 PCGs of *M. stejnegeri* are calculated, revealing a high A+T content in the whole mitogenome. The two rRNA genes, 12S rRNA and 16S rRNA, are 996 bp and 1602 bp in length, respectively. The sequences of the 22 tRNA genes range from 66 bp (*trnC* and *trnS1*) to 76 bp (*trnW*) and can be folded into a cloverleaf secondary structure with normal base pairing, except for *trnS1*, which has lost the entire dihydrouridine arm. This study illustrates the mitogenome characteristics of *M. stejnegeri*, and the results may be useful in understanding both the taxonomy of species within the *Melanitta* genus and in developing potential molecular markers for phylogenetic analyses. Based on all acquired sequence data, the phylogenetic relationships of 76 Anseriformes species were reconstructed. The results suggest that *M. stejnegeri* is a sister group to *M. deglandi*, and five *Melanitta* species form a closely related *Melanitta* clade. The results also indicate that *L. specularioides* and *S. specularis* are sister groups. Further study is required to elucidate their phylogenetic relationship with other avian species. However, inconsistencies in morphological characteristic classification and molecular phylogenetic relationships exist among four avian species: *A. capensis*, *A. georgica*, *S. rhynchotis*, and *S. smithii*. Additionally, it was discovered for the first time that *C. hyemalis* and *S. mollissima* are sister groups. Their dietary habits may exert similar selective pressures on their mitochondrial genomes. Previous phylogenetic analyses based solely on mitochondrial genomes may have resulted in skewed phylogenetic relationships. Future studies may necessitate the utilization of whole-genome data and a broader range of specimens to achieve a more reliable classification of avian species.

## Figures and Tables

**Table 1:** Composition and skew values for *M. stejnegeri*.

*M. stejnegeri*	Size (bp)	A%	C%	T%	G%	A+T%	G+C%	AT-Skew	GC-Skew
mtDNA	16,631	28.72	33.52	21.57	16.20	50.29	49.71	0.1422	−0.3483
PCGs	11,407	26.25	34.64	22.84	16.28	49.08	50.92	0.0695	−0.3605
tRNA	1546	29.11	22.32	26.39	22.19	55.50	44.50	0.0490	−0.0029
rRNA	2598	33.26	27.02	19.36	20.36	52.62	47.38	0.2641	−0.1405
D-loop	1071	26.89	33.05	23.81	16.25	50.70	49.30	0.0608	−0.3409

## Data Availability

The genome sequence data that support the findings of this study are openly available in GenBank of NCBI at https://www.ncbi.nlm.nih.gov (accessed on 8 July 2024), under the accession no. PP990569.

## Supplementary Materials

The following supporting information can be downloaded at: https://www.mdpi.com/article/10.3390/ijms251810181/s1.

## References

[1] Sun S.E., Li Q., Kong L.F., Yu H. (2016). Complete mitochondrial genomes of *Trisidos kiyoni* and *Potiarca pilula*: Varied mitochondrial genome size and highly rearranged gene order in Arcidae. Sci. Rep..

[2] Boore J.L. (1999). Animal mitochondrial genomes. Nucleic Acids Res..

[3] Pacheco M.A., Battistuzzi F.U., Lentino M., Aguilar R.F., Kumar S., Escalante A.A. (2011). Evolution of modern birds revealed by mitogenomics Timing the radiation and origin of major orders. Mol. Biol. Evol..

[4] Qian L.F., Wang H., Yan J., Pan T., Jiang S.Q., Rao D.Q., Zhang B.W. (2018). Multiple independent structural dynamic events in the evolution of snake mitochondrial genomes. BMC Genom..

[5] Zhou X.P., Lin Q.X., Fang W.Z., Chen X.L. (2014). The complete mitochondrial genomes of sixteen ardeid birds revealing the evolutionary process of the gene rearrangements. BMC Genom..

[6] BirdLife International, Species Factsheet: Siberian Scoter Melanitta stejnegeri. https://datazone.birdlife.org/species/factsheet/siberian-scoter-melanitta-stejnegeri.

[7] Gill F., Donsker D., Rasmussen P. IOC World Bird List (v14.1), 2024. https://www.worldbirdnames.org/new/.

[8] Chen P., Li J.Q., Li H.B., Lu Q., Liu W., Zhang J.L. (2023). Characterization of the complete mitochondrial genome of sea duck *Mergus serrator* and comparison with other Anseriformes species. Pak. J. Zool..

[9] Sun Z.L., Pan T., Hu C.C., Sun L., Ding H.W., Wang H., Zhang C.L., Jin H., Chang Q., Kan X.Z. (2017). Rapid and recent diversification patterns in Anseriformes birds: Inferred from molecular phylogeny and diversification analyses. PLoS ONE.

[10] Yuan Z.F., Liu P., Lu X., Zhu D., Liu J., Guo Q., Zhang W.P., Duan Y.B. (2024). Complete mitochondrial genome and phylogenetic analysis of the blue whistling thrush (*Myophonus caeruleus*). Genes.

[11] Hassanin A., Léger N., Deutsch J. (2005). Evidence for multiple reversals of asymmetric mutational constraints during the evolution of the mitochondrial genome of Metazoa, and consequences for phylogenetic inferences. Syst. Biol..

[12] Liu D.W., Zhou Y.W., Fei Y.L., Xie C.P., Hou S.L. (2021). Mitochondrial genome of the critically endangered Baer’s Pochard, *Aythya baeri*, and its phylogenetic relationship with other Anatidae species. Sci. Rep..

[13] Ojala D., Montoya J., Attardi G. (1981). tRNA punctuation model of RNA processing in human mitochondria. Nature.

[14] Chen L., Lin Y.F., Xiao Q., Lin Y., Du Y., Lin C.X., Ward-Fear G., Hu C.C., Qu Y.F., Li H. (2021). Characterization of the complete mitochondrial genome of the many-lined sun skink (*Eutropis multifasciata*) and comparison with other Scincomorpha species. Genomics.

[15] Pereira S.L. (2000). Mitochondrial genome organization and vertebrate phylogenetics. Genet. Mol. Biol..

[16] Steinberg S., Cedergren R. (1994). Structural compensation in atypical mitochondrial tRNAs. Nat. Struct. Biol..

[17] Kuhle B., Hirschi M., Doerfel L.K., Lander G.C., Schimmel P. (2022). Structural basis for shape-selective recognition and aminoacylation of a D-armless human mitochondrial Trna. Nat. Commun..

[18] Biela A., Hammermeister A., Kaczmarczyk I., Walczak M., Koziej L., Lin T.Y., Glatt S. (2023). The diverse structural modes of tRNA binding and recognition. J. Biol. Chem..

[19] Sun C.H., Liu H.Y., Min X., Lu C.H. (2020). Mitogenome of the little owl *Athene noctua* and phylogenetic analysis of Strigidae. Int. J. Biol. Macromol..

[20] Ruokonen M., Kvist L. (2002). Structure and evolution of the avian mitochondrial control region. Mol. Phylogenet. Evol..

[21] Yang B., Zhang Z., Yang C.Q., Wang Y., Orr M.C., Wang H., Zhang A.B. (2022). Identification of species by combining molecular and morphological data using convolutional neural networks. Syst. Biol..

[22] Zou Z.T., Zhang J.Z. (2016). Morphological and molecular convergences in mammalian phylogenetics. Nat. Commun..

[23] Liu G., Zhou L.Z., Li B., Zhang L.L. (2014). The complete mitochondrial genome of *Aix galericulata* and *Tadorna ferruginea* Bearings on their phylogenetic position in the Anseriformes. PLoS ONE.

[24] Zhang W.P., Qiu B.Y., Zhang D.S. (2023). Adaptive evolution analysis of mitochondrial genomes in Anseriform birds with various feeding habits. Curr. Biotechnol..

[25] Andrews S. FastQC A Quality Control Tool for High Throughput Sequence Data. https://www.bioinformatics.babraham.ac.uk/projects/fastqc/.

[26] Meng G.L., Li Y.Y., Yang C.T., Liu S.L. (2019). MitoZ A toolkit for animal mitochondrial genome assembly, annotation and visualization. Nucleic. Acids. Res..

[27] Bernt M., Donath A., Jühling F., Externbrink F., Florentz C., Fritzsch G., Pütz J., Middendorf M., Stadler P.F. (2013). MITOS Improved de novo metazoan mitochondrial genome annotation. Mol. Phylogenet. Evol..

[28] Lowe T.M., Chan P.P. (2016). tRNAscan-SE On-line Integrating search and context for analysis of transfer RNA genes. Nucleic Acids Res..

[29] Kerpedjiev P., Hammer S., Hofacker I.L. (2015). Forna (force-directed RNA): Simple and effective online RNA secondary structure diagrams. Bioinformatics.

[30] Tamura K., Stecher G., Kumar S. (2021). MEGA11 Molecular evolutionary genetics analysis version 11. Mol. Biol. Evol..

[31] Perna N.T., Kocher T.D. (1995). Patterns of nucleotide composition at fourfold degenerate sites of animal mitochondrial genomes. J. Mol. Evol..

[32] Grant J.R., Enns E., Marinier E., Mandal A., Herman E.K., Chen C.Y., Graham M., Van Domselaar G., Stothard P. (2023). Proksee In depth characterization and visualization of bacterial genomes. Nucleic Acids Res..

[33] Xia X., Xie Z. (2001). DAMBE: Software package for data analysis in molecular biology and evolution. J. Hered..

[34] Ronquist F., Teslenko M., van der Mark P., Ayres D.L., Darling A., Höhna S., Larget B., Liu L., Suchard M.A., Huelsenbeck J.P. (2012). MrBayes 3.2: Efficient bayesian phylogenetic inference and model choice across a large model space. Syst. Biol..

[35] Nylander J. (2004). MrModeltest V2. Program distributed by the author. Bioinformatics.

[36] Darriba D., Taboada G.L., Doallo R., Posada D. (2012). jModelTest 2 More models, new heuristics and parallel computing. Nat. Methods.

[37] Rambaut A. FigTree v.1.4.4. http://tree.bio.ed.ac.uk/software/figtree/.

