# Supplementary material for: The Complete Mitochondrial Genome of the Siberian Scoter Melanitta stejnegeri and Its Phylogenetic Relationship in Anseriformes
Source: Int J Mol Sci. 2024 Sep 22;25(18):10181. doi: 10.3390/ijms251810181 (PMC11432269; doi:10.3390/ijms251810181)
Supplement: Supplementary file 1 [file ijms-25-10181-s001.zip › Supplementary Materials.pdf]

Table S1. Annotation of the mitochondrial genome of *M. stejnegeri*.

Gene	Nucleotide No.		Size(bp)	Codon		Spacer/ Overlap (-)	Strand
	From	To		Start	Stop		
D-loop	1	1071	1071				H
trnF	1072	1142	71			-1	H
12S rRNA	1142	2137	996			0	H
trnV	2138	2208	71			0	H
16S rRNA	2209	3810	1602			0	H
trnL2	3811	3884	74			6	H
ND1	3891	4868	978	ATG	AGG	-2	H
trnI	4867	4939	73			7	H
trnQ	4947	5017	71			-1	L
trnM	5017	5085	69			0	H
ND2	5086	6124	1039	ATG	T	0	H
trnW	6125	6200	76			3	H
trnA	6204	6272	69			1	L
trnN	6274	6346	73			0	L
trnC	6347	6412	66			0	L
trnY	6413	6482	70			1	L
COX1	6484	8034	1551	GTG	AGG	-9	H
trnS2	8026	8098	73			2	L
trnD	8101	8169	69			1	H
COX2	8171	8857	688	GTG	TAA	1	H
trnK	8859	8927	69			1	H
ATP8	8929	9096	168	ATG	TAA	-10	H
ATP6	9087	9770	684	ATG	TAA	-1	H
COX3	9770	10553	784	ATG	T	0	H
trnG	10554	10622	69			0	H
ND3	10623	10974	352	ATG	TAA	1	H
trnR	10976	11045	70			-1	H
ND4L	11046	11342	297	ATG	TAA	-7	H
ND4	11336	12713	1378	ATG	T	0	H
trnH	12714	12782	69			0	H
trnS1	12783	12848	66			-1	H
trnL1	12848	12918	71			-1	H
ND5	12919	14742	1824	GTG	TAA	-1	H
CYTB	14742	15884	1143	ATG	TAA	2	H
trnT	15887	15955	69			7	H
trnP	15963	16032	70			9	L
ND6	16042	16563	522	ATG	TAG	0	L
trnE	16564	16631	68				L

Table S2. Codon number and relative synonymous codon usage (RSCU) for the mitochondrial protein-coding genes (PCGs) of *M. stejnegeri*.

Codon	Count	RSCU	Codon	Count	RSCU	Codon	Count	RSCU	Codon	Count	RSCU
UUU(F)	42	0.38	UCU(S)	23	0.5	UAU(Y)	23	0.45	UGU(C)	3	0.21
UUC(F)	177	1.62	UCC(S)	94	2.06	UAC(Y)	79	1.55	UGC(C)	26	1.79
UUA(L)	37	0.33	UCA(S)	83	1.82	UAA(*)	7	2.8	UGA(W)	83	1.57
UUG(L)	14	0.13	UCG(S)	18	0.39	UAG(*)	1	0.4	UGG(W)	23	0.43
CUU(L)	42	0.38	CCU(P)	17	0.29	CAU(H)	11	0.2	CGU(R)	5	0.27
CUC(L)	181	1.62	CCC(P)	107	1.82	CAC(H)	101	1.8	CGC(R)	15	0.8
CUA(L)	285	2.55	CCA(P)	91	1.55	CAA(Q)	69	1.52	CGA(R)	37	1.97
CUG(L)	111	0.99	CCG(P)	20	0.34	CAG(Q)	22	0.48	CGG(R)	18	0.96
AUU(I)	48	0.35	ACU(T)	28	0.36	AAU(N)	8	0.13	AGU(S)	5	0.11
AUC(I)	223	1.65	ACC(T)	159	2.02	AAC(N)	112	1.87	AGC(S)	51	1.12
AUA(M)	104	1.22	ACA(T)	115	1.46	AAA(K)	74	1.66	AGA(*)	0	0
AUG(M)	67	0.78	ACG(T)	13	0.17	AAG(K)	15	0.34	AGG(*)	2	0.8
GUU(V)	32	0.65	GCU(A)	31	0.37	GAU(D)	7	0.22	GGU(G)	15	0.27
GUC(V)	66	1.33	GCC(A)	193	2.33	GAC(D)	57	1.78	GGC(G)	78	1.4
GUA(V)	71	1.43	GCA(A)	86	1.04	GAA(E)	72	1.52	GGA(G)	74	1.33
GUG(V)	29	0.59	GCG(A)	22	0.27	GAG(E)	23	0.48	GGG(G)	56	1

Table S3. Base composition of ribosomal and transfer RNA genes of *M. stejnegeri*.

	T%	C%	A%	G%	Size(bp)	A+T%	G+C%
12S rRNA	18.67	29.22	31.53	20.58	996	50.20	49.80
16S rRNA	19.79	25.66	34.33	20.22	1602	54.12	45.88
Avg	19.36	27.02	33.26	20.36	1299.00	52.62	47.38
trnF	12.68	30.99	33.80	22.54	71	46.48	53.52
trnV	21.13	28.17	29.58	21.13	71	50.70	49.30
trnL2	21.62	28.38	27.03	22.97	74	48.65	51.35
trnI	21.92	24.66	32.88	20.55	73	54.79	45.21
trnQ	39.44	9.86	29.58	21.13	71	69.01	30.99
trnM	20.29	33.33	27.54	18.84	69	47.83	52.17
trnW	25.00	25.00	34.21	15.79	76	59.21	40.79
trnA	36.23	14.49	28.99	20.29	69	65.22	34.78
trnN	28.77	17.81	27.40	26.03	73	56.16	43.84
trnC	24.24	22.73	16.67	36.36	66	40.91	59.09
trnY	37.14	17.14	20.00	25.71	70	57.14	42.86
trnS2	30.14	16.44	23.29	30.14	73	53.42	46.58
trnD	23.19	23.19	33.33	20.29	69	56.52	43.48
trnK	23.19	27.54	31.88	17.39	69	55.07	44.93
trnG	27.54	23.19	33.33	15.94	69	60.87	39.13
trnR	22.86	27.14	30.00	20.00	70	52.86	47.14
trnH	26.09	23.19	34.78	15.94	69	60.87	39.13
trnS1	22.73	27.27	25.76	24.24	66	48.48	51.52
trnL1	25.35	22.54	32.39	19.72	71	57.75	42.25
trnT	28.99	18.84	34.78	17.39	69	63.77	36.23
trnP	35.71	11.43	27.14	25.71	70	62.86	37.14
trnE	26.47	17.65	25.00	30.88	68	51.47	48.53
Avg	26.39	22.32	29.11	22.19	70.27	55.50	44.50

Table S4. Accession numbers of 76 Anseriformes species and one outgroup.

Family	Genus	Species name	Data sources	Accession number	Mitogenome	Genome
Anatidae	*Aix*	*Aix galericulata*	Mitogenome	NC_023969, GCA_024635365.1	√	√
		A. sponsa	Genome, nucleotides	GCA_032270905, EU585668.1, AF173741.1	NA	√
	Anas	*Anas acuta*	Mitogenome	NC_024631, GCA_963932015.1	√	√
		A. bernieri	Genome, nucleotides	GCA_032165215, AF059121.1	NA	√
		A. capensis	Genome	GCA_032357685	NA	√
		A. chathamica	Mitogenome	KF562761	√	NA
		A. crecca	Mitogenome	KF203133, GCA_036873605.1	√	√
		A. georgica	Genome	GCA_032275265	NA	√
		A. platyrhynchos	Mitogenome	MW354666, GCA_015476345.1	√	√
		A. poecilorhyncha	Mitogenome	KF156760, GCA_040966495.1	√	√
		A. zonorhyncha	Mitogenome	MZ593724, GCA_002224875.1	√	√
	Anser	*Anser albifrons*	Mitogenome	NC_004539	√	NA
		A. anser	Mitogenome	NC_011196, GCA_964211835.1	√	√
		A. brachyrhynchus	Mitogenome	OZ035927.1, GCA_002592135.1	√	√
		A. cygnoides	Mitogenome	NC_023832, GCA_040182565.1	√	√
		A. fabalis	Mitogenome	HQ890328	√	NA
		A. indicus	Mitogenome	NC_025654, GCA_025583725.1	√	√
	Asarcornis	*Asarcornis scutulata*	Mitogenome	NC_052827, GCA_013398475.1	√	√
	Aythya	*Aythya americana*	Mitogenome	NC_000877	√	NA
		A. baeri	Mitogenome	NC_057048, GCA_026413565.1	√	√
		A. ferina	Mitogenome	NC_024602, GCA_964211825.1	√	√
		A. fuligula	Mitogenome	OQ979258, GCA_009819795.1	√	√
		A. marila	Mitogenome	NC_068663, GCA_029042245.1	√	√
		A. nyroca	Mitogenome	NC_059801	√	NA
	Bucephala	*Bucephala albeola*	Mitogenome	MW849286	√	NA
		B. clangula	Mitogenome	MW849283, GCA_964059595.1	√	√
		B. islandica	Mitogenome	MW849281	√	NA
	Branta	*Branta bernicla*	Mitogenome	KJ680301	√	NA
		B. canadensis	Mitogenome	NC_007011, GCA_006130075.1	√	√
		B. hutchinsii	Genome, nucleotides	GCA_032270845, FJ582576.1	NA	√
	Cairina	*Cairina moschata*	Mitogenome	NC_010965, GCA_018104995.1	√	√
	Clangula	*Clangula hyemalis*	Mitogenome	MW077850, GCA_963989345.1	√	√
	Cygnus	*Cygnus atratus*	Mitogenome	NC_012843, GCA_013377495.2	√	√

	C. buccinator	Genome, nucleotides	GCA_019232035, AY666404.1, AY509690.1, EF165358.1	NA	√
	C. columbianus bewickii	Mitogenome	NC_017604	√	NA
	C. cygnus	Mitogenome	KP981363, GCA_014362685.1	√	√
	C. olor	Mitogenome	NC_027096, GCA_009769625.2	√	√
Dendrocygna	*Dendrocygna javanica*	Mitogenome	NC_012844	√	NA
Heteronetta	*Heteronetta atricapilla*	Genome	GCA_011075105	NA	√
Histrionicus	*Histrionicus histrionicus*	Mitogenome	MW849288	√	NA
Lophodytes	*Lophodytes cucullatus*	Mitogenome	MW849287	√	NA
Lophonetta	*Lophonetta specularioides*	Genome	GCA_032165235	NA	√
Mareca	*Mareca americana*	Mitogenome	NC_088449.1, GCA_035588595.1	√	√
	M. falcata	Mitogenome	MW020580, GCA_038382085.1	√	√
	M. penelope	Mitogenome	MW020582, GCA_036026335.1	√	√
	M. sibilatrix	Genome, nucleotides	GCA_034783215, FJ027105.1, EU914149.1, AF059168.1	NA	√
	M. strepera	Mitogenome	NC_045373, GCA_035583915.1	√	√
Melanitta	*Melanitta americana*	Mitogenome	MW849282	√	NA
	M. deglandi	Mitogenome	MW849279	√	NA
	M. fusca	Genome	GCA_029620185	NA	√
	M. perspicillata	Mitogenome	MW849280	√	NA
	M. stejnegeri	Mitogenome	PP990569	√	NA
Mergus	*Mergus merganser*	Mitogenome	NC_040986	√	NA
	M. serrator	Mitogenome	MZ365040	√	NA
	M. squamatus	Mitogenome	NC_016723	√	NA
Netta	*Netta rufina*	Mitogenome	NC_024922, GCA_964035555.1	√	√
Nettapus	*Nettapus auritus*	Genome	GCA_011076525	NA	√
Oxyura	*Oxyura jamaicensis*	Mitogenome	MW574354, GCA_011077185.1	√	√
Polysticta	*Polysticta stelleri*	Mitogenome	MW849289	√	NA
Sibirionetta	*Sibirionetta formosa*	Mitogenome	NC_015482, GCA_038406215.1	√	√
Somateria	*Somateria fischeri*	Mitogenome	MW849290	√	NA
	S. mollissima	Mitogenome	MW849292, GCA_951411735.1	√	√
	S. spectabilis	Mitogenome	MW849291	√	NA
Spatula	*Spatula clypeata*	Mitogenome	KT345702, GCA_038406095.1	√	√
	S. discors	Genome	GCA_032165275	NA	√
	S. querquedula	Genome, nucleotides	GCA_032191525, AF059146.1	NA	√
	S. rhynchotis	Genome	GCA_034783165	NA	√
	S. smithii	Genome	GCA_035221675	NA	√

	Speculanas	*Speculanas specularis*	Genome, nucleotides	GCA_032165255, AF059150.1	NA	√
	Stictonetta	*Stictonetta naevosa*	Genome	GCA_011074415	NA	√
	Tachyeres	*Tachyeres brachypterus*	Genome	GCA_032357605	NA	√
		T. leucocephalus	Genome	GCA_034780855	NA	√
	Tadorna	*Tadorna ferruginea*	Mitogenome	NC_024640	√	NA
		T. tadorna	Mitogenome	KU140668	√	NA
Anseranatidae	*Anseranas*	*Anseranas semipalmata*	Mitogenome	MN356217, GCA_013399115.1	√	√
Anhimidae	*Chauna*	*Chauna torquata*	Mitogenome	NC_052807, GCA_013399475.1	√	√
Phasianidae	*Gallus*	*Gallus gallus*	Mitogenome	MH732978.1, GCA_018104995.1	√	√
