# Supplementary figures and images for: The Complete Mitochondrial Genome of the Siberian Scoter Melanitta stejnegeri and Its Phylogenetic Relationship in Anseriformes
Source: Int J Mol Sci. 2024 Sep 22;25(18):10181. doi: 10.3390/ijms251810181 (PMC11432269; doi:10.3390/ijms251810181)

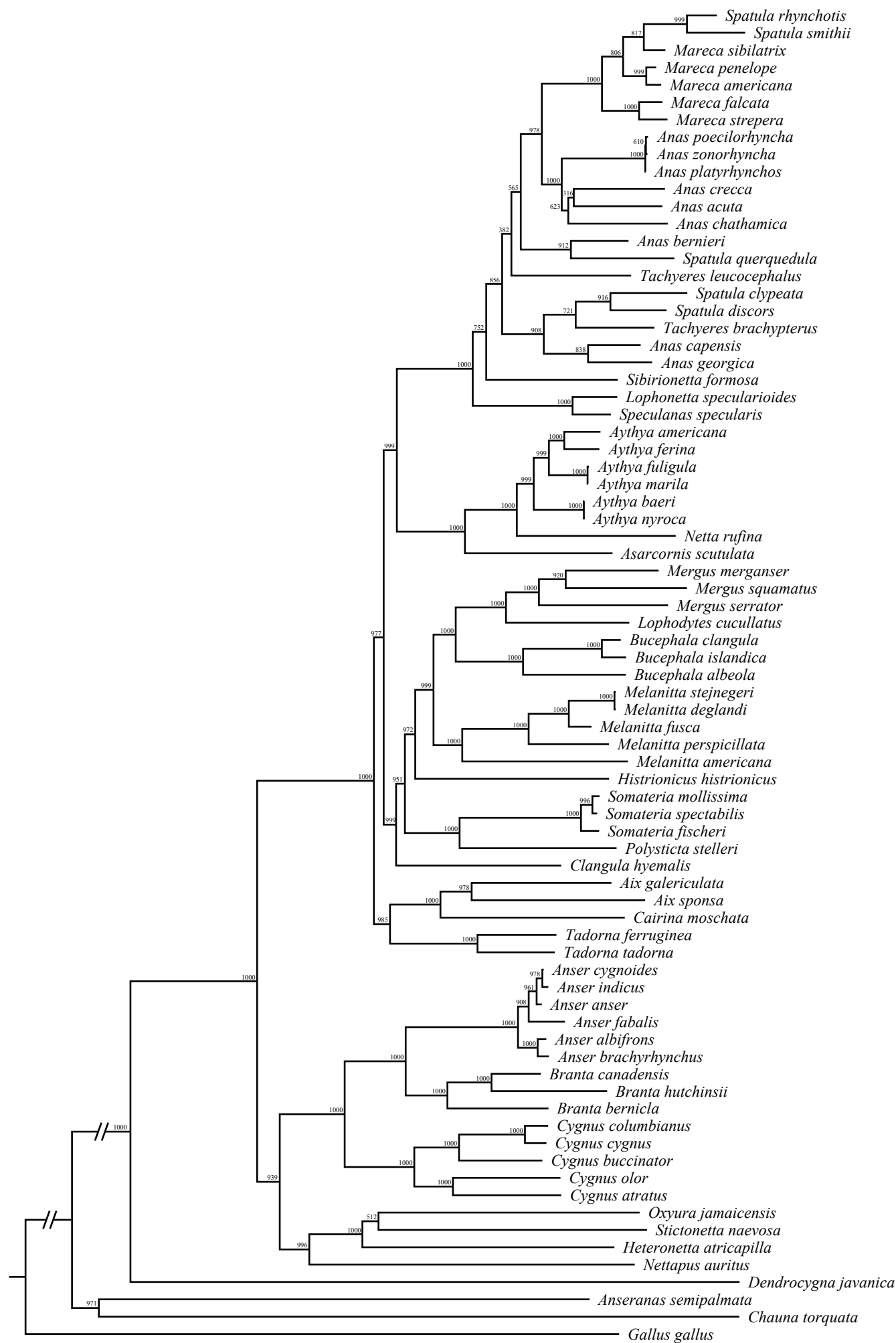

Anatidae

Anseranatidae  
Anhimidae  
Phasianidae

Supplement: Supplementary file 1 [file ijms-25-10181-s001.zip › Figure S1_Phylogenetic relationships of 76 Anseriformes species reconstructed using the Maximum Likelihood (ML) method based on 13 mitochondrial protein-c.pdf]
